# Preservation of Multiple Mammalian Tissues to Maximize Science Return from Ground Based and Spaceflight Experiments

**DOI:** 10.1371/journal.pone.0167391

**Published:** 2016-12-01

**Authors:** Sungshin Choi, Hami E. Ray, San-Huei Lai, Joshua S. Alwood, Ruth K. Globus

**Affiliations:** 1 KBRwyle, Moffett Field, California, United States of America; 2 ASRC Federal Space and Defense, Inc., Moffett Field, California, United States of America; 3 Space Biosciences Division, NASA-Ames Research Center, Moffett Field, California, United States of America; University of Palermo, ITALY

## Abstract

**Background:**

Even with recent scientific advancements, challenges posed by limited resources and capabilities at the time of sample dissection continue to limit the collection of high quality tissues from experiments that can be conducted only infrequently and at high cost, such as in space. The resources and time it takes to harvest tissues post-euthanasia, and the methods and duration of long duration storage, potentially have negative impacts on sample quantity and quality, thereby limiting the scientific outcome that can be achieved.

**Objectives:**

The goals of this study were to optimize methods for both sample recovery and science return from rodent experiments, with possible relevance to both ground based and spaceflight studies. The first objective was to determine the impacts of tissue harvest time post-euthanasia, preservation methods, and storage duration, focusing on RNA quality and enzyme activities in liver and spleen as indices of sample quality. The second objective was to develop methods that will maximize science return by dissecting multiple tissues after long duration storage *in situ* at -80°C.

**Methods:**

Tissues of C57Bl/6J mice were dissected and preserved at various time points post-euthanasia and stored at -80°C for up to 11 months. In some experiments, tissues were recovered from frozen carcasses which had been stored at -80°C up to 7 months. RNA quantity and quality was assessed by measuring RNA Integrity Number (RIN) values using an Agilent Bioanalyzer. Additionally, the quality of tissues was assessed by measuring activities of hepatic enzymes (catalase, glutathione reductase and GAPDH).

**Results:**

Fresh tissues were collected up to one hour post-euthanasia, and stored up to 11 months at -80°C, with minimal adverse effects on the RNA quality of either livers or RNAlater-preserved spleens. Liver enzyme activities were similar to those of positive controls, with no significant effect observed at any time point. Tissues dissected from frozen carcasses that had been stored for up to 7 months at -80°C had variable results, depending on the specific tissue analyzed. RNA quality of liver, heart, and kidneys were minimally affected after 6–7 months of storage at -80°C, whereas RNA degradation was evident in tissues such as small intestine, bone, and bone marrow when they were collected from the carcasses frozen for 2.5 months.

**Conclusion:**

These results demonstrate that 1) the protocols developed for spaceflight experiments with on-orbit dissections support the retrieval of high quality samples for RNA expression and some protein analyses, despite delayed preservation post-euthanasia or prolonged storage, and 2) many additional tissues for gene expression analysis can be obtained by dissection even following prolonged storage of the tissue *in situ* at -80°C. These findings have relevance both to high value, ground-based experiments when sample collection capability is severely constrained, and to spaceflight experiments that entail on-orbit sample recovery by astronauts.

## Introduction

Challenges continue to exist in regards to acquiring high quality samples from animal tissues despite recent technical advances. From the time spent collecting the samples to the use of stabilizing or preserving agents, and ultimately to the storage conditions and duration prior to actual analysis, each step is critical and requires attention. Timing plays a key role in obtaining high quality samples. Ideally, samples are harvested immediately post-euthanasia by trained and experienced staff, and the time between death and sample stabilization is minimized to limit sample degradation. If the samples are not analyzed at the time of euthanasia, then additional factors that may affect sample quality are of concern, including freezing rate and duration of storage. Understanding these challenges faced in the laboratory aids the researcher to optimize protocols to maximize sample quality and accomplish science objectives; translating those practices for use in the space environment, however, raises new challenges.

Several factors potentially affect sample quality, and ultimately, the downstream experimental results. The procedure to obtain high quality RNA varies between different laboratories [1]. The most reliable source for obtaining high quality RNA is by purification from freshly dissected tissues [2–7]. A factor to be taken into consideration when tissues are not processed immediately at the time of harvesting is the time samples are exposed to periods of warm ischemia, which may occur during and after surgery, and between sample thawing and processing. In some instances, warm ischemia-induced degradation alters gene expression profiles and RNA quality in certain tissues, although the process appears to have selective effects on individual genes [4, 8, 9].

RNA is subject to degradation by ubiquitous RNAses, and high quality RNA is needed for analysis of gene expression, next-generation sequencing, gene profiling and other “Omics” analyses in the study of molecular mechanisms. The addition of an RNA stabilization solution to tissues, either by immersion or perfusion, has become standard practice for RNA stabilization [7, 10, 11]. Studies where select tissues are frozen immediately upon harvesting, with or without stabilization buffer, show no significant detriment to the quality of the RNA [6, 12–16]. Frozen tissues that are allowed to thaw at room temperature up to 16 hours show significant degradation in samples without a stabilization buffer such as RNAlater, versus those stored in RNAlater [14]. Recent work published by Gupta et al describe retention of RNA quality in rodent male and female reproductive organs when samples are placed into RNAlater up to 40 minutes after euthanasia and in RNAlater treated samples stored at -80°C for 10 months [17]. Other studies reveal severe degradation of RNA in frozen tissues when allowed to thaw for up to 30 minutes prior to analysis, suggesting the necessity for immediate processing of certain tissues [18, 19].

In the studies reported here, we investigated various time points and storage conditions for retrieving tissues from mice, and then determined the impact that these conditions had on sample quality. By measuring RNA Integrity Number (RIN) and enzyme activities in tissues under various recovery scenarios, we have characterized and established an acceptable timeline for rodent tissue isolation, preservation, and storage to optimize science return from both ground based and on-orbit flight sample collection.

## Materials and Methods

### Animals

Adult female C57Bl/6J mice from Jackson Laboratory (Bar Harbor, ME) were used in all the experiments. All animals were euthanized by intraperitoneal injection of Euthasol (Virbac, Fort Worth, TX) followed by cervical dislocation. All efforts were made to minimize suffering. The Institutional Animal Care and Use Committee at the NASA Ames Research Center approved the animal care and experimental procedures.

### Tissue collection

**Fig 1:**
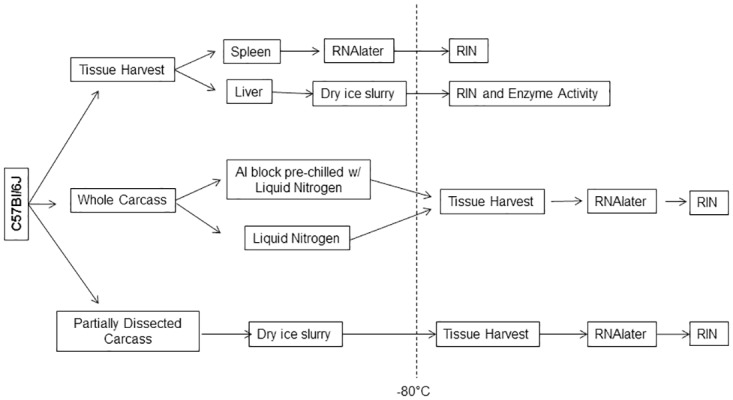
Experimental design schematic. Tissues were harvested at various times from C57Bl/6J mice post-euthanasia (fresh spleen and liver) or from frozen whole or partially dissected carcasses. Samples and carcasses were stored at -80°C for various durations as long as 11 months prior to tissue harvest (carcasses) and analysis of liver enzyme activities or RNA quality (RIN), as described in Methods.

Fresh tissue samples: To provide positive control tissues representing preservation by standard laboratory conditions, spleens and livers were harvested immediately post-euthanasia then were either promptly (within 5 min) frozen in liquid nitrogen (liver) or preserved in RNAlater (spleen). To simulate the anticipated dissection timing by astronauts on-orbit, spleens were removed at 6 or 22 minutes after euthanasia, and livers were removed at 9 or 25 minutes post-euthanasia (Fig 1), then placed in cryovials (Thermo Fisher Scientific, Waltham, MA). Spleens were preserved in RNAlater (Thermo Fisher Scientific, Waltham, MA) and livers were frozen by embedding the cryovials in pelleted dry ice. Samples were either stored at -80°C for 3.5 mo (spleen) and 4.5 mo (liver), or for 11 mo prior to analysis.

Time-delay between euthanasia and stabilization: To bracket the possible time frames that the astronaut crew may need to dissect then preserve the samples, spleens were harvested at 1, 20, 40, 60, 80 and 100 minutes post-euthanasia and preserved in RNAlater. Livers were harvested from the same mice at 2, 25, 45, 65, 85, and 105 minutes post-euthanasia and frozen in dry ice. Samples were stored at -80°C for 1 month prior to analysis.

Whole Carcass (WC): Mice were euthanized by intraperitoneal injection of Euthasol followed by cervical dislocation. The carcasses were then wrapped in two layers of foil and placed in Ziploc bags. The carcasses were frozen using an aluminum block that was pre-chilled with liquid nitrogen, to simulate the freezing kit used on-orbit, and transferred to a -80°C freezer for storage (up to 10 months). Various tissues (spleen, liver, heart, kidney, lung, brain, adrenal glands, eye, thymus, small intestine, large intestine, femur, tibia, hindlimb muscle, and bone marrow) were collected from 2.5, 4 and 6–7 month frozen WC after thawing at room temperature for 20–30 minutes, and then preserved in RNAlater. Positive control WC were immersed in liquid nitrogen immediately after euthanasia and were stored in a 80°C freezer for 4 and 6–7 months.

Partially Dissected Carcass (PC): Spleens and livers were removed following euthanasia, and utilized for a different study. The dissected carcasses then were wrapped with two layers of aluminum foil, and sealed in a Ziploc bag before freezing on dry ice. The time from euthanasia to the carcass freezing in dry ice was about 2 hours. PC were stored at -80°C for 2.5 months prior to harvesting the tissues (brain, adrenal glands, eye, thymus, small intestine, hindlimb muscle, and bone marrow). PC were removed from the -80°C freezer, and placed at room temperature with the aluminum foils open for partial thawing to the extent that dissection commenced, which was up to 35 minutes after removal from -80°C. Tissues were preserved in RNAlater.

### RNA isolation and analysis

RNA was extracted from homogenized tissues using TRIzol reagents (Thermo Fisher Scientific, Waltham, MA). RNA concentration and purity were assessed using a NanoDrop 2000 UV-Vis Spectrophotometer (Thermo Fisher Scientific, Waltham, MA). RNA quality was measured by calculating the RNA integrity number (RIN) using the Bioanalyzer 2100 (Agilent Technologies, Santa Clara, CA), where a RIN of 10 is considered intact and 0 totally degraded RNA [20, 21]. The analysis was performed according to the manufacturer’s instructions.

### Liver enzyme assays

The activities of three hepatic enzymes (catalase, glutathione reductase and GAPDH) were measured to assess sample quality after various collection conditions. Catalase activity was measured using the colorimetric assay for Catalase (Oxford Biomedical Research, Oxford, MI) according to the manufacturer’s instructions, and normalized to total protein levels (Thermo Scientific^™^ Pierce^™^ Micro BCA^™^ Protein Assay Kit, Pierce Biotechnology, Rockford, IL). Catalase protein levels were measured using the Catalase Human ELISA Kit (Abcam, Cambridge, MA), and specific activity of the enzyme calculated (U/μg catalase protein). Glutathione reductase activity levels were determined using the Glutathione Reductase (GR) Assay Kit (Abcam, Cambridge, MA) and GAPDH activity levels were determined using the colorimetric GAPDH assay kit (ScienCell Research Laboratories, Carlsbad, CA). The activity levels were also normalized by the total protein concentrations using the Micro BCA Protein Assay Kit. Positive controls were obtained according to standard laboratory protocols where dissections were followed by immersion of the samples in liquid nitrogen.

### Statistical analysis

Statistical analysis was performed using either parametric or non-parametric tests. The data sets were first assessed for normality using the Shapiro-Wilk test, where a p-value <0.05 indicated non-normally distributed data. If the data were normally distributed, one way ANOVA followed by Tukey's post hoc test was performed. For non-normally distributed data, non-parametric tests were performed using the Kruskal-Wallis test and Dunn post-hoc test (Joint Ranks, compared to Control). In non-parametric boxplots, medians within the interquartile (boxes) and full range (whiskers) are displayed. p<0.05 was considered significant throughout.

## Results

### RNA quality in tissues collected at various time points post-euthanasia

**Fig 2:**
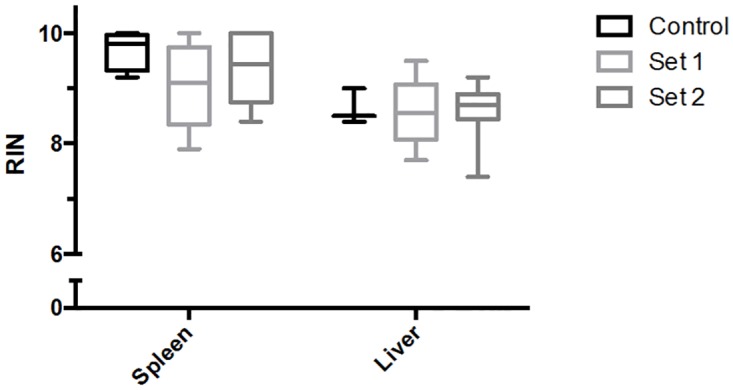
Effects of preservation times post-euthanasia on RNA quality in spleen and liver. Spleens and livers were collected from up to 3 minutes (Positive Control), 9 minutes (Set 1), and 25 minutes (Set 2) post-euthanasia. Set 1 and 2 time points were selected to simulate anticipated dissection timing in microgravity. Spleens were preserved in RNAlater, and livers were frozen on dry ice. All samples were stored at -80°C, and analyzed after 3.5 months for spleen and 4.5 months for liver. RNA quality was measured by calculating the Bioanalyzer-based RNA integrity number (RIN) using the Bioanalyzer 2100. Samples harvested up to 25 minutes post-euthanasia, and stored for less than 5 months at -80°C, yielded RIN values greater than 8. Data sets were assessed for normality using the Shapiro-Wilk test, followed by the Kruskal-Wallis test. Values shown are medians within interquartile (boxes) and full range (whiskers). Spleens: n = 4, 9, and 8 for Control, Set 1 and 2, respectively. Livers: n = 3, 10, and 10 for Control, Set 1 and 2, respectively.

**Fig 3:**
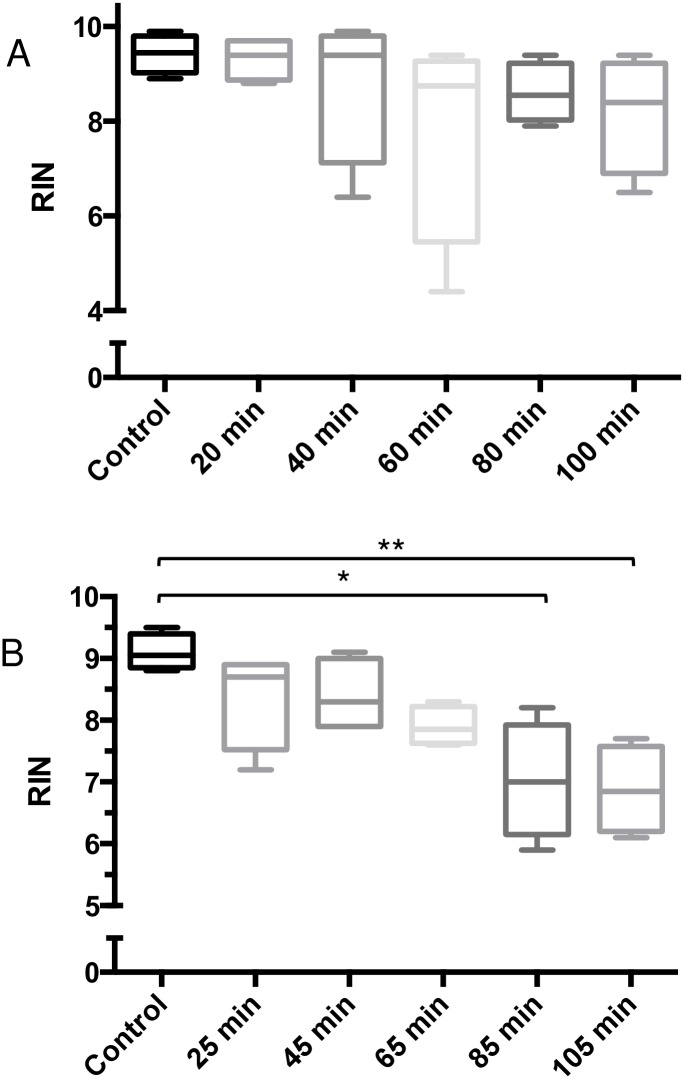
Effects of delayed dissections on RNA quality in spleens and livers. Spleens were collected at 1 (Positive Control), 20, 40, 60, 80, and 100 minutes post-euthanasia, and were preserved in RNAlater. RNAlater-preserved spleen samples yielded high RIN values (RIN > 7.5) for all time points (A). Liver tissues from the same mice in (A) were collected at 2 (Positive Control), 25, 45, 65, 85, and 105 minutes post-euthanasia, and were frozen on dry ice. RNA quality in samples collected up to 45 minutes post-euthanasia were minimally affected, resulting in RIN values > 8. RIN value decreased by 24% in samples collected at 105 minutes post-euthanasia, compared to controls (B). All samples were stored at -80°C for 1 month prior to analysis. Data sets were assessed for normality using the Shapiro-Wilk test, followed by the Dunn post-hoc test (Joint Ranks, compared to Control). Values shown are medians within interquartile (boxes) and full range (whiskers). (n = 4 for each time point). * p<0.05, ** p<0.01

To determine if harvest times post-euthanasia alters RNA quality, freshly-dissected spleens and livers were isolated at various time points, preserved as shown in Fig 1 and Table 1, and stored at -80°C for less than 5 months prior to RNA purification. RNA purified from spleens and livers that were dissected up to 25 minutes post-euthanasia and stored at -80°C for 3.5 and 4.5 months, respectively, showed no significant detrimental effect on the quality of the RNA (RIN > 8), when compared to the control samples (Fig 2). Although spleen samples collected up to 100 minutes post-euthanasia, and preserved in RNA later, also showed that RNA quality was minimally affected (RIN > 8) (Fig 3a), liver samples showed a significant decline in RNA quality after collection times of 45 minutes (RIN < 8 at 65 minutes, RIN < 7 at 85 minutes and RIN < 6.8 at 105 minutes) (Fig 3b). Together, these data demonstrated that spleens can be collected up to 100 minutes after euthanasia to yield high quality RNA (RIN > 8) when preserved in RNAlater (stored at -80°C). In contrast, a significant decline in liver RIN values was observed 65 minutes after euthanasia, when compared to control samples.

**Table 1:** Summary of RIN values from harvested spleen and liver samples.

**Sample**	**RIN**	**Storage at -80°C (months)**	**Collection Time**
Spleen preserved in RNAlater	> 8	1	≤100 minutes
	≥ 8.5	3.5	≤25 minutes
	≥ 8.5	11	≤5 minutes
Liver frozen on dry ice	≥ 7	1	≤85 minutes
	≥ 8.5	4.5	≤25 minutes
	≥ 8.5	11	≤5 minutes
**Sample**	**RIN**	**Storage at -80°C (months)**	**Collection Time After Removal from -80°C Freezer**
WC: spleen dissected after carcass storage at -80°C	> 5	2.5	≤5 minutes
	> 5	4	≤5 minutes
	> 4	6 to 7	≤5 minutes
WC: liver dissected after carcass storage at -80°C	> 6.5	2.5	≤5 minutes
	> 8	4	≤5 minutes
	> 7.5	6 to 7	≤5 minutes

**Fig 4:**
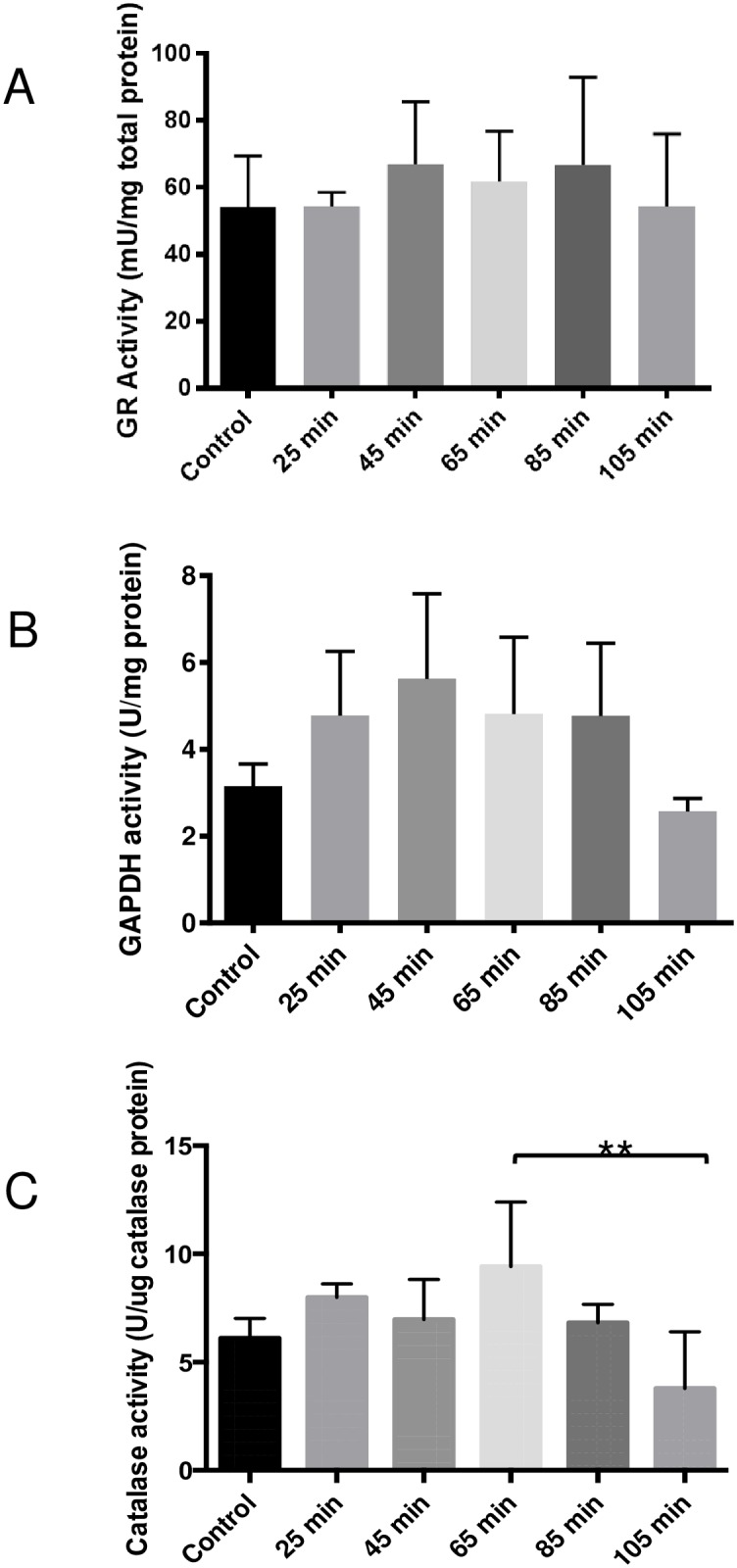
Effects of time-delayed dissections on hepatic enzyme levels. Activities of three hepatic enzymes, glutathione reductase, GAPDH and catalase, were measured from samples collected at various times post-dissection. Glutathione reductase (A) and GAPDH (B) activity was similar to those of positive controls, with no significant effects observed at any time point. Catalase activities were similar to those of the positive controls; however, samples collected at 105 minutes post-euthanasia displayed significantly lower levels when compared to tissue collected at 65 minutes (C). Activity levels of glutathione reductase and GAPDH were normalized by the total protein concentrations. Catalase activity levels were normalized by catalase protein. Data sets were assessed for normality using the Shapiro-Wilk test, followed by the one-way ANOVA and Tukey’s post hoc test. Values are means ± SD (n = 4). ** p<0.01

Activity levels of three liver enzymes also were measured from the time-delayed dissections. Glutathione reductase (Fig 4a) and GAPDH (Fig 4b) levels were similar to those of the positive controls. Catalase activities were only moderately lower in the samples collected at 105 minutes after dissection compared to the control samples (Fig 4c). Overall, the activities of these enzymes in harvested liver tissues were not significantly affected by collection time or storage at -80°C for 1 month.

### Effect of storage duration at -80°C on tissue quality

**Fig 5:**
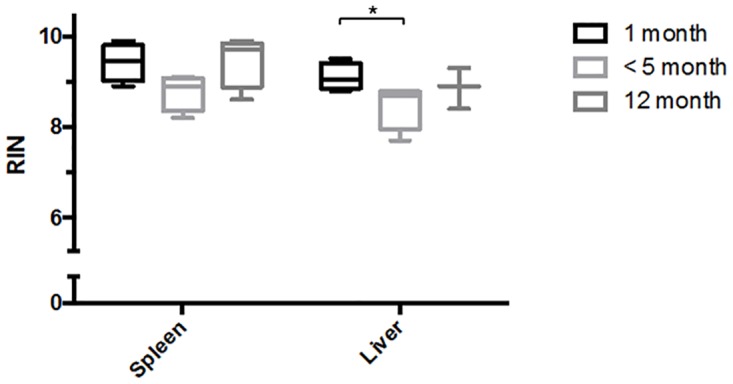
Effects of long duration storage at -80°C on RNA quality in spleen and liver. Spleens and livers were collected up to 5 minutes post-euthanasia. Spleens were preserved in RNAlater, and livers were frozen on dry ice. Samples were stored at -80°C for 1, 3.5, and 11 months for spleen, and 1, 4.5, and 11 months for liver. Samples stored up to 11 months at -80°C resulted in RIN values greater than 8. Data sets were assessed for normality using the Shapiro-Wilk test, followed by the Kruskal-Wallis test. Values shown are medians within interquartile (boxes) and full range (whiskers) (n = 4 for all months except for n = 3 for Liver 12 months). * p<0.05

To assess the influence of storage duration on sample quality, spleens and livers were collected per standard laboratory procedures (up to 5 minutes post-euthanasia), and then stored at -80°C for 1, 3.5, 4.5, and 11 months prior to analysis. At all time points investigated, no significant degradation was found in the RNA quality of any of the tissue samples (RIN > 8) (Fig 5). These data demonstrate that quality of the tissues can be maintained for up to 11 months when the tissues are stored at -80°C.

### Integrity of RNA harvested from whole frozen carcasses

**Fig 6:**
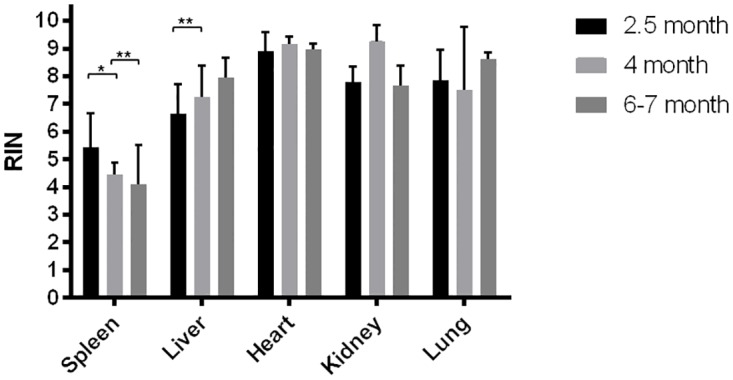
Integrity of RNA harvested from whole frozen carcasses. Whole frozen carcasses (WC) were frozen using an aluminum block that was pre-chilled with liquid nitrogen to simulate the freezing kit used on-orbit (2.5, 4 and 6–7 month WC) prior to being placed into a -80°C freezer. Spleen, liver, heart, kidney, and lung were collected within 30 minutes post thawing of the WC, and preserved in RNAlater prior to analysis. Data sets were assessed for normality using the Shapiro-Wilk test, followed by the one-way ANOVA and Tukey’s post hoc test. Values are means ± SD (n = 9 for 2.5 month, except n = 8 for lung; n = 4, 5, 6, 3, 4 for 4 month spleen, liver, heart, kidney and lung, respectively; n = 3 for 6–7 month). * p<0.05; ** p<0.01

To assess RNA quality from whole frozen carcasses (WC), we analyzed the spleen, liver, heart, kidney, and lung dissected from WC that were stored at -80°C for 2.5, 4, and 6–7 months. Results from spleens showed that increased duration at -80°C from 2.5 to 6–7 months caused a 25% decline in RIN values. Long durations of storage (4-7months) had minimal effects on RNA quality of liver, heart, kidney, and lung although RIN values were 6% lower when comparing WC liver at 6–7 months (Fig 6) to fresh liver which was dissected first then frozen for 11 months at -80°C prior to analysis (Fig 5).

**Fig 7:**
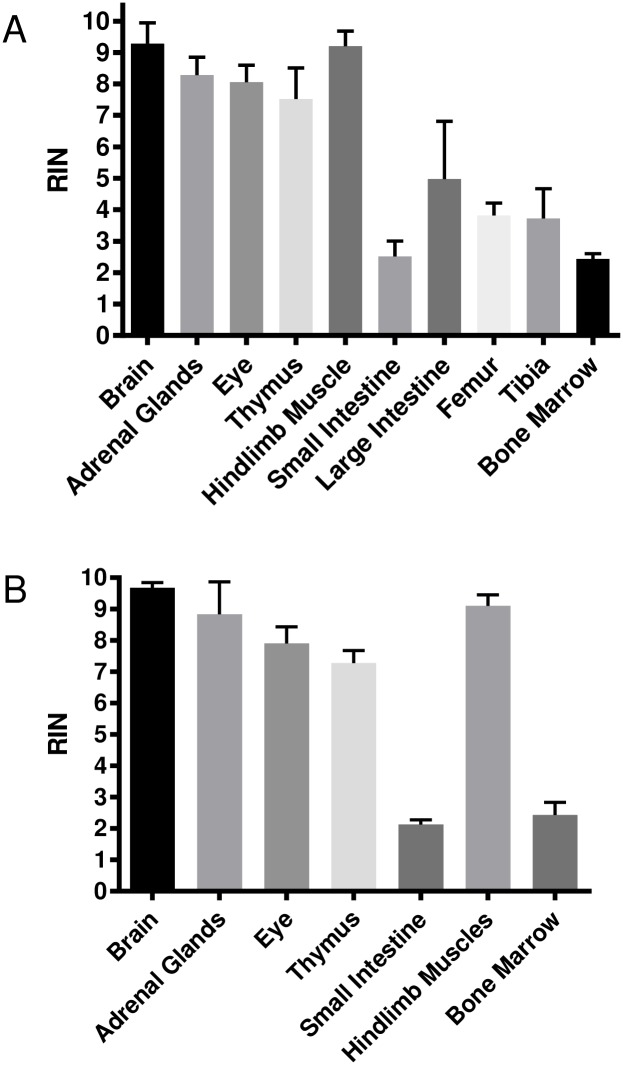
The effects on RNA quality in various tissues recovered from whole and partially dissected carcasses stored at -80°C for 2.5 months. Tissue samples were removed from the frozen carcasses for RIN analysis. Tissues were preserved in RNAlater prior to analysis. Brain, adrenal glands, eye, thymus, and hindlimb muscles resulted in high RIN values in both WC (A) and PC (B). RNA quality in WC and PC small intestine and bone marrow samples were severely degraded, resulting in RIN values < 3 (A and B). Values are means ± SD (n = 8 for Fig 7A, except n = 7 adrenal gland, n = 5 femur and bone marrow, and n = 4 tibia; n = 4 for Fig 7B, except n = 3 adrenal gland).

Using a separate group of WC we dissected numerous additional tissues and determined the RNA quality. High quality RNA was recovered from various tissues (brain, adrenal glands, eye, thymus and hindlimb muscle); RIN values were greater than 7.5 in all tissues (Fig 7a). Small intestine and bone marrow, however, showed severe degradation (RIN values less than 3,) despite rapid preservation in RNAlater at the time of dissection. Additionally, femur, large intestine, and tibia yielded RNA with RIN values less than 5 (Fig 7a). These findings suggest that after long term storage in -80C high quality RNA can be recovered from select tissues of whole carcasses.

### Integrity of RNA harvested from partially dissected carcasses

To determine if the carcasses that were stored after select tissue dissection also can yield high quality samples of other tissue types after long-term storage, tissues were harvested from partially dissected carcasses stored at -80°C for 2.5 months. Similar to the results found as in WC; PC tissue samples resulted in high RNA quality recovered from brain, adrenal glands, eye, thymus and hindlimb muscles. Small intestine and bone marrow samples showed severe degradation similar to that seen in WC (Fig 7b).

## Discussion

Our results demonstrated that high quality RNA (RIN> 7) could be isolated from most of the tissues, even from tissues stored for a prolonged period of time (results summarized in Table 1). However, RNA samples isolated from select tissues dissected from frozen carcasses (e.g. spleen, small intestine, bone marrow and mineralized tissues) showed degradation even with short duration storage, probably due to high endogenous RNase activity.

Tissues preserved in RNAlater showed significant improvement in RNA quality compared to tissues immersed in liquid nitrogen [22]. In the present study, we observed that freshly harvested spleens preserved in RNAlater showed a higher RIN (RIN >8) compared to spleens dissected from frozen carcasses (RIN = ~5.5). In the absence of RNAlater, liver samples yielded relatively high quality RNA (RIN >6.5).

Sample quality collected under various conditions varied depending on tissue type and endpoint measured. Spleens collected up to 100 minutes after euthanasia yielded high quality RNA since they were preserved in RNAlater. Liver tissues collected up to 65 minutes after euthanasia, also yielded high quality samples. However, liver samples showed a significant decline in RNA quality (RIN <8) after 65 minutes post-euthanasia. These data demonstrate that tissues collected with appropriate timing that is typical for laboratory dissections (i.e., up to 30 minutes between euthanasia and tissue collection) can yield samples of acceptable quality to achieve exceptional science return, as expected [23]. These findings are consistent with those of others who showed that RNA samples from tonsils can be stable for several hours, and even after overnight storage at room temperature, RNA did not significantly degrade [6]. However, long-term storage (e.g. 16 hours) at room temperature could result in significant changes in expression levels of some genes. In addition to the time delay in tissue collection and storage, the repetitive freezing and thawing of the snap-frozen tissues could affect RNA integrity. However, RNA degradation in frozen liver tissue did not occur in seconds or over few minutes, but over 30 minutes of thawing at room temperature [18]. Thus, if tissues accidentally are thawed for a brief period, those tissues do not necessarily have to be discarded from gene expression analyses. Schoor et al. report that partially degraded RNA may still be usable even though suboptimal quality [24]. Furthermore, it was reported that RNA with low RIN still allows for reliable monitoring of mRNA expression by qPCR if small amplicon sizes are targeted, although large amplicons failed to generate measurable expression values [25]. These findings, along with our own, allow investigators in the future to use carcass that have been preserved by freezing, to address questions that may arise at a later time.

Testing methods in ground based experiments by applying anticipated on-orbit scenarios, reduces the risk of recovering samples of inadequate quality from high cost spaceflight experiments. Spleens and livers collected at various time points post-euthanasia to simulate challenging conditions for dissections in microgravity yielded samples with high RIN values, thus anticipating return of samples with high quality from space even if preservation post-euthanasia is delayed up to 1 hour. When plant samples were placed into a -20°C freezer, severe RNA degradation resulted; however, when the plant samples were preserved in RNAlater and stored at either 4°C or -20°C, the integrity of RNA was maintained. Furthermore, wheat samples preserved in RNAlater from an experiment performed on the ISS, yielded RNA quality comparable to fresh samples, even after undergoing fluctuations of storage temperatures [26]. More recently, RNAlater preserved plant samples were shown to yield sufficient amounts of protein to allow broad-scale proteomics analyses [27].

We showed that multiple tissues collected from frozen carcasses yielded high quality RNA, which could be applied to RNAseq, gene profiling and qPCR. High quality sample recovery for RNA and at least some enzymes is possible from most tissues, despite either delayed preservation post-euthanasia or prolonged storage. Based on our RIN results, it is our recommendation that the collection of tissue samples occur within 45 minutes for studies requiring high quality RNA and within 85 minutes for usual RNA studies. Should sample retrieval occur from frozen carcasses, our studies show that many, but not all tissues yield high RNA quality up to 7 months storage time at -80°C.

## Supporting Information

S1 File Supplemental File for Data Availability. Data sets for this study are fully available without restriction.
(PDF)
