# Supplementary material for: Preservation of Multiple Mammalian Tissues to Maximize Science Return from Ground Based and Spaceflight Experiments
Source: PLoS One. 2016 Dec 1;11(12):e0167391. doi: 10.1371/journal.pone.0167391 (PMC5132293; doi:10.1371/journal.pone.0167391)
Supplement: S1 File — Data sets for this study are fully available without restriction. (PDF) [file pone.0167391.s001.pdf]

Fig 2	Control	Control	Control	Control	Set 1	Set 1	Set 1
Spleen	9.9	9.2	10	9.7	8.1	9.1	9.6
Liver	8.5	9	8.4		9	9.3	8.4

Fig 3A		Control	Control	20 min	20 min	40 min	40 min
Spleen		9.4		9.1		9.5	
		9.5		9.7		6.4	
		9.9		8.8		9.3	
		8.9		9.7		9.9	

Fig 3B		Control	Control	25 min	25 min	45 min	45 min
Liver		8.8		7.2		7.9	
		9.5		8.9		9.1	
		9		8.5		8.7	
		9.1		8.9		7.9	

Fig 4A	Control	Control	25 min	25 min	45 min	45 min	65 min
GR		48.47264		56.35644		49.21743	
		43.69352		55.46981		76.3321	
		76.91982		47.95436		88.6154	
		47.42072		57.33481		53.61765	

Fig 4B	Control	Control	25 min	25 min	45 min	45 min	65 min
GAPDH		3.688		3.872		2.77	
		2.562		4.37		5.943	
		3.446		3.891		7.15	
		2.901		6.974		6.62	

Fig 4C		Control	Control	25 min	25 min	45 min	45 min
Catalase		6.06		7.33		5.21	
		4.88		8.46		5.72	
		7.03		7.57		9.2	
		6.45		8.6		7.75	

Fig 5	1 month	1 month	1 month	1 month	1 month	1 month	< 5 month
Spleen		9.4	9.5	9.9	8.9		
Liver		8.8	9.5	9	9.1		

Fig 6	2.5 month	2.5 month	2.5 month	2.5 month	2.5 month	2.5 month	2.5 month
Spleen		5.9	6.2	3.7	7.3	6.8	4.8

Liver			6.3	8.4	8	6.4	6.4	4.9
Heart			8.7	9.2	9.2	9.6	9.8	8.3
Kidney			7.9	9.1	7.8	7.2	7.8	7.5
Lung			6	7.1	8	9.5	7.6	8.9

Fig 7A		Brain	Brain	Adrenal Gla	Adrenal Gla	Eye	Eye	Thymus
tissues			8.5		8.6		7.2	
			9.2		8.5		8.4	
			9.9		7.4		8.7	
			10		8.4		8.4	
			8.1		7.6		8.6	
			9.4		8.6		7.7	
			9.7		8.9		7.8	
			9.4				7.6	

Fig 7B		Brain	Brain	Adrenal Gla	Adrenal Gla	Eye	Eye	Thymus
		9.5		8.5		8		7.7
		9.7				7.2		7.1
		9.6		8		8.5		6.8
		9.9		10		7.9		7.5

Set 1	Set 1	Set 1	Set 1	Set 1	Set 1	Set 1	Set 2	Set 2
8.6	9.5	7.9	9	10	9.9		10	8.4
8.1	8.9	8.5	9.5	8	7.7	8.6	8.8	8.7

60 min	60 min	80 min	80 min	100 min	100 min
4.4		8.7		8.7	
8.9		8.4		9.4	
9.4		7.9		6.5	
8.6		9.4		8.1	

65 min	65 min	85 min	85 min	105 min	105 min
8		8.2		6.5	
7.7		5.9		7.2	
7.6		6.9		7.7	
8.3		7.1		6.1	

65 min	85 min	85 min	105 min	105 min
66.3549		50.67123		51.86097
45.51899		45.13035		42.01345
55.1506		67.41564		37.88998
80.3922		103.4816		85.51857

65 min	85 min	85 min	105 min	105 min
3.288		4.358		2.169
3.943		2.577		2.882
4.734		6.203		2.676
7.309		5.941		2.546

65 m	65 m	85 min	85 min	105 min	105 min
9.66		6.35		1.35	
7.57		6.35		2.17	
6.92		6.47		7.23	
13.52		8.09		4.37	

< 5 month	< 5 month	< 5 month	< 5 month	< 5 month	12 month	12 month	12 month	12 month
9.1	8.8	8.2	9			9.7	8.6	9.7
8.7	8.8	7.7	8.7			8.4	8.9	9.3

2.5 month	2.5 month	2.5 month	4 month	4 month	4 month	4 month	4 month	4 month
4.4	4.4	5.6		4.3	4.1	4.3	5.1	

7	6.7	5.6		5.6	6.7	7.6	8.3	8.1
8.3	7.7	9.3		9.3	9.2	9.5	8.7	9.2
7.3	7.8	7.6		9.5	9.7	8.6		
7.5	8.3			8.9	8.5	8.5	4.1	

Thymus	Hindlimb M	Hindlimb Muscle			Small Intes	Small Intes	Large Intes	Large Intes
8.1		8.6				2.6		6.3
6.1		9.3				2.7		3
7.3		9.6				2.1		2.7
8.2		9.7				2.3		7.6
6.4		8.4				2		6.3
7.2		9.6				3.6		4.1
9.1		9.1				2.4		3.6
7.8		9.3				2.4		6.2

Thymus	Small Intes	Small Intes	Hindlimb M	Hindlimb M	Bone Marr	Bone Marrow
	2.2		9.6		2.3	
	2		9.1		1.9	
	2		8.8		2.7	
	2.3		8.9		2.8	

Set 2	Set 2	Set 2	Set 2	Set 2	Set 2	Set 2	Set 2
9.6	10	9.3	8.7	8.9	10		
8.5	7.4	8.7	9.2	8.6	8.3	8.9	8.9

12 month	12 month
9.9	

4 month	4 month	4 month	4 month	6-7 month	6-7 month	6-7 month	6-7 month	6-7 month
					4.1	2.7	5.5	

					7.9	8.7	7.3
9					8.8	8.9	9.2
					7.8	6.9	8.3
					8.9	8.5	8.5

Femur	Femur	Tibia	Tibia	Bone Marr	Bone Marrow
	3.3		4.9		2.4
	4.1		4		2.5
	4.1		2.7		2.7
	4.1		3.3		2.3
	3.5				2.3

6-7 month 6-7 month 6-7 month 6-7 month 6-7 month
